# Capsids and Genomes of Jumbo-Sized Bacteriophages Reveal the Evolutionary Reach of the HK97 Fold

**DOI:** 10.1128/mBio.01579-17

**Published:** 2017-10-17

**Authors:** Jianfei Hua, Alexis Huet, Carlos A. Lopez, Katerina Toropova, Welkin H. Pope, Robert L. Duda, Roger W. Hendrix, James F. Conway

**Affiliations:** a Department of Structural Biology, University of Pittsburgh School of Medicine, Pittsburgh, Pennsylvania, USA; b Department of Biological Sciences, University of Pittsburgh, Pittsburgh, Pennsylvania, USA; Columbia University College of Physicians & Surgeons

**Keywords:** bacteriophage, capsid, cryo-EM, evolution, genome, HK97, jumbophage, phage

## Abstract

Large icosahedral viruses that infect bacteria represent an extreme of the coevolution of capsids and the genomes they accommodate. One subset of these large viruses is the jumbophages, tailed phages with double-stranded DNA genomes of at least 200,000 bp. We explored the mechanism leading to increased capsid and genome sizes by characterizing structures of several jumbophage capsids and the DNA packaged within them. Capsid structures determined for six jumbophages were consistent with the canonical phage HK97 fold, and three had capsid geometries with novel triangulation numbers (T=25, T=28, and T=52). Packaged DNA (chromosome) sizes were larger than the genome sizes, indicating that all jumbophages use a head-full DNA packaging mechanism. For two phages (PAU and G), the sizes appeared very much larger than their genome length. We used two-dimensional DNA gel electrophoresis to show that these two DNAs migrated abnormally due to base modifications and to allow us to calculate their actual chromosome sizes. Our results support a ratchet model of capsid and genome coevolution whereby mutations lead to increased capsid volume and allow the acquisition of additional genes. Once the added genes and larger capsid are established, mutations that restore the smaller size are disfavored.

## IMPORTANCE

A large family of viruses share the same fold of the capsid protein as bacteriophage HK97, a virus that infects bacteria. Members of this family use different numbers of the capsid protein to build capsids of different sizes. Here, we examined the structures of extremely large capsids and measured their DNA content relative to the sequenced genome lengths, aiming to understand the process that increases size. We concluded that mutational changes leading to larger capsids become locked in by subsequent changes to the genome organization.

## INTRODUCTION

The capsid is a critical and complex part of a virus’s structure, as it must contain and protect the viral chromosome. Since each capsid is built from multiple copies of only a few kinds of protein subunits, an elaborate process is required to orchestrate the initial assembly and the large-scale conformational changes that accompany capsid maturation. Additional factors are required to package the viral chromosome and allow the attachment of the cell recognition and entry apparatus, which will later effect release of the chromosome into a new host. Although the capsids of double-stranded DNA (dsDNA) tailed phages and herpesviruses share the canonical fold of phage HK97 (1), their capsid sizes vary considerably. Smaller phage capsids, such as those of the well-known phages λ and T7, are ~500 Å in diameter, whereas the massive phage G capsid is 1,500 to 1,800 Å in diameter with an internal volume for accommodating viral dsDNA that is ~10-fold greater than for the T=7 capsids (2). Such larger capsids, however, occupy a poorly explored corner of the biological world, and only a few have been characterized structurally: ΦKZ (3), ΦRSL1 (4), and ΦM19 (5). Nonetheless, they lead to fundamental questions about biological size determination and mechanisms of virus evolution, with potential benefits in learning how virus assembly can be derailed and how viruses acquire novel capabilities.

**FIG 1:**
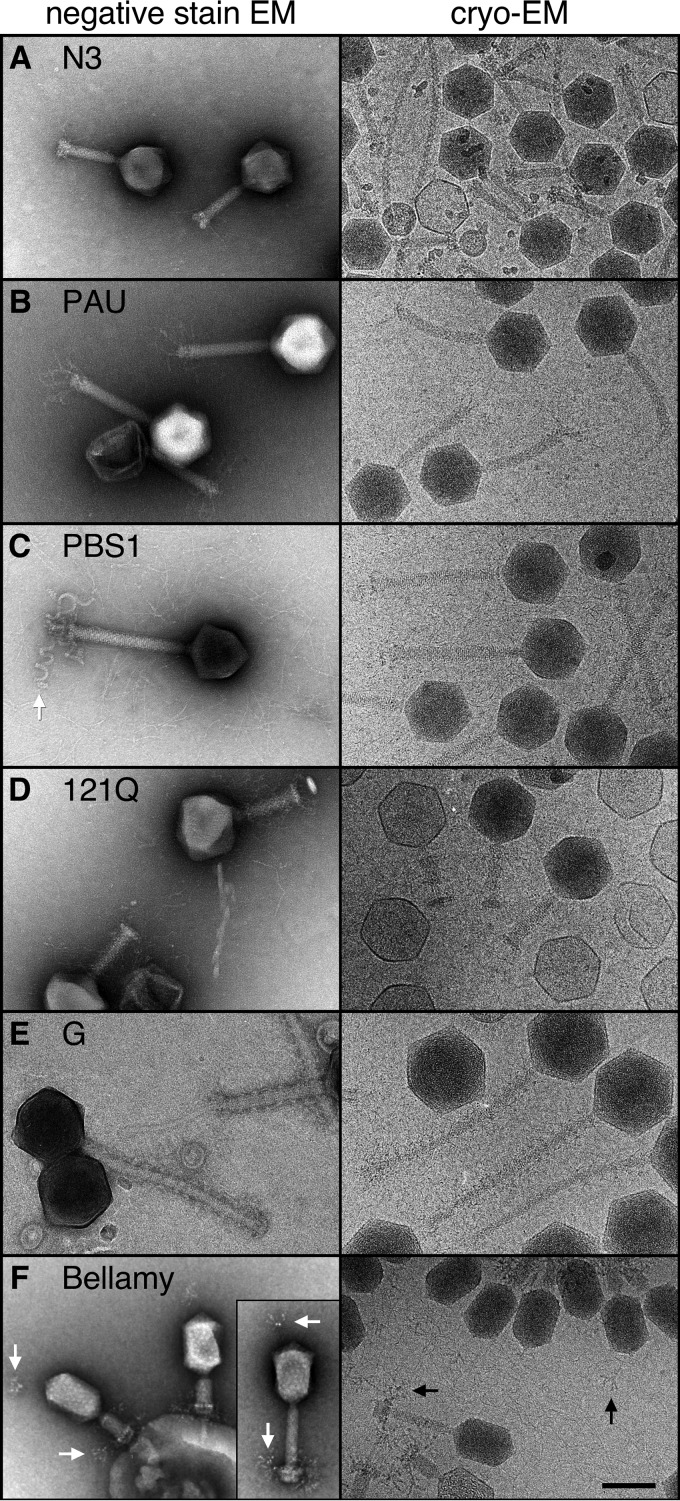
Electron microscopy of phage particles. Representative portions from negative-stain (left) and cryo-EM (right) images are shown for the phage particles selected for this study. All have icosahedral capsids except for phage Bellamy (F), which is prolate icosahedral, and all tails are contractile. Note the unusual corkscrew-shaped fibers attached to the tail baseplate of PBS1 (arrow in C) that adsorb to the flagella of *Bacillus subtilis* (14). Also unusual is the fiber cluster attached to the head of phage Bellamy that closely resembles those on the baseplate (arrows in F). Bar, 100 nm.

Our focus is on the interactions between capsid structure, determined from cryo-electron microscopy (cryo-EM) and biochemistry, and genetic structure of the packaged DNA and how together these influence the biological properties of the virus. We describe here our studies of six large icosahedral capsids (Fig. 1) with diameters ranging from 1,000 to 1,800 Å, including some of the largest viruses known, which we term “jumbophages” (6). Four of the six phages assemble capsids with icosahedral geometries not previously seen. We investigated the occurrence of “inner body” structures by using so-called bubblegrams (7), and we present the first measurements of DNA packing densities in these large capsids via a technique we developed for accurately assessing the DNA size when it is substantially modified.

## RESULTS

### Phages, hosts, and sources.

The jumbophages in this report were isolated from a variety of environments and hosts. The *Synechococcus* sp. phage Bellamy was isolated from the harbor of Jamestown, RI, and *Sinorhizobium meliloti* phage N3 and *Bacillus subtilis* phage PBS1 were both isolated from soil in Ottawa, Canada. N3 and PBS1 have been used as generalized transducing phages in common *Rhizobium* strains and in *Bacillus subtilis*, respectively (8–10). PAU was isolated from samples of diseased silkworms (*Bombyx mori* L.) collected in Karnataka State, India, and found to be active in *Pseudomonas paucimobilis* (11). The *Escherichia coli* phage 121Q was isolated from sewage samples in Romania (12). Phage G and its host, *Bacillus megaterium*, were isolated in Italy (2).

### Virion morphologies.

The six phages discussed here share the property that their genomes are >200 kbp, our *ad hoc* defining characteristic of a “jumbo” phage. They also share the head-and-tail morphology of the viral order *Caudovirales*, the tailed dsDNA phages. Figure 1 shows negative-stain and cryo-electron micrograph images of the virions. All six phages have contractile tails; this appears to be the most common tail morphology in known examples of jumbophages. A closer look at the 121Q and G virions showed that there are long fibers around the capsid and tail sheath of 121Q, and there are short fibers around the sheath of phage G. Bellamy has branched fibers that appear to be attached to the top of the head and to the baseplate. PBS1 particles have short fibers extending from the sheath and thick corkscrew-shaped helical tail fibers, described previously (13), and appear to mediate adsorption to host flagella (14).

### Capsid morphologies.

**FIG 2:**
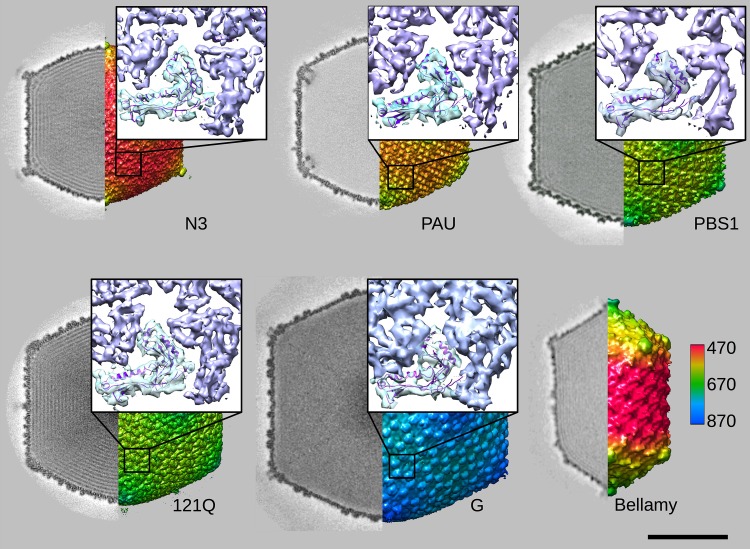
Quality assessment of the jumbophage density maps. Surface views of EM capsid reconstructions for each jumbophage, colored by radius length (in angstroms) (see color key on lower left). Sections through each map are shown on the left, and fits of the HK97 major capsid protein into the maps are shown in the insets. Note that the PAU density map was calculated from empty capsids that were significantly more abundant than DNA-filled phage particles, and these revealed inner structures beneath the vertices that will be discussed in a future manuscript. Bar, 50 nm.

**FIG 3:**
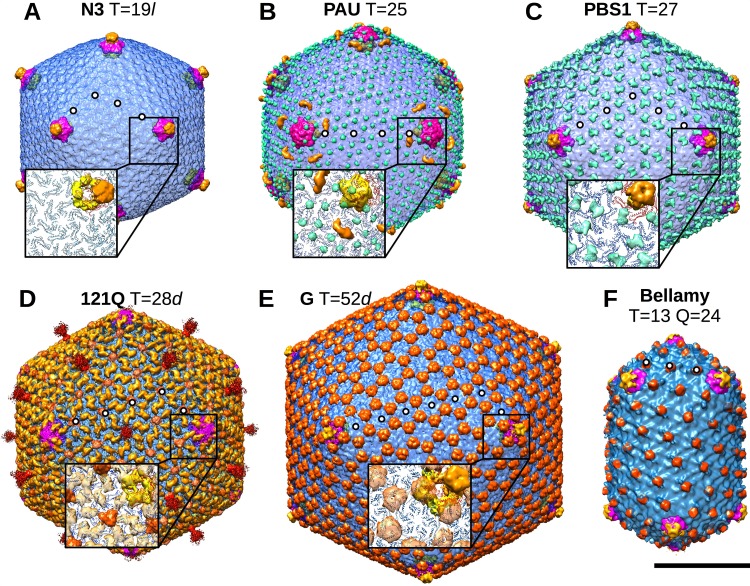
Surface views of capsid reconstructions calculated from cryo-EM data. Phage names and triangulation numbers are indicated. Insets show portions of models derived from the HK97 major capsid protein atomic structure (PDB ID 2FT1) fit into the cryo-EM density maps, except for Bellamy, for which the resolution was too poor for confident fitting. Density encompassed by the hexamer fits is shown in blue, and that encompassed by the pentamer fit is shown in purple. Additional unfit density on the capsid exterior is green when it appeared to belong to the major capsid protein and yellow, orange, or red when the density corresponded to a putative decoration protein. Additional density located inside the capsid under the five-fold vertices is shown in yellow in the insets. White dots indicate the indices for assigning T numbers, e.g., in panel A, h = 3, k = 2, and therefore T = 3^2^ + 3.2 + 2^2^, or T=19, for phage N3 (see Table 2). Assignment of handedness was inferred by the fits. Bar, 50 nm.

Surface views and cross-sections of the density maps calculated for the six phage capsids are shown in Fig. 2 and 3. Five of the six capsids have the polyhedral shape typical of mature dsDNA phages and one is prolate. All have recognizable hexamer and pentamer arrangements, and all appear to include decoration proteins bound to the exterior surface. Triangulation numbers (T) are readily assigned based on capsomer organization, ranging from T=19 for N3 to the massive G phage capsid with T=52. The prolate Bellamy capsid has T=13 end caps, in common with phage T4, but it has a larger elongation number (Q) (15): Q=24 for Bellamy, compared to Q=20 for phage T4 (16). Three of the six capsids have T numbers not seen before, suggesting that with sufficient sampling of the environment it may be possible to find structures of all possible values of T according to the following equation (17): T = h^2^ + (h × k) + k^2^, with h = 1, 2, 3,… and k = 0, 1, 2, 3,…, up to an as-yet-unknown limit of capsid size. Capsids with T-numbers up to 16, i.e., T=1, 3, 4, 7, 9, 12, 13, or 16, have been generally well documented, but only a few capsids beyond that range have been characterized, i.e., T=19, 21, 25, 27, 28, 31, 36, 37, 39, 43, 48, 49, 52, and beyond. Phage G (T=52) has the largest icosahedral virus capsid documented for a phage, although some algal viruses are as large (18–21), and the *Mimiviridae* family viruses are considerably larger (22, 23).

Although the large capsid sizes impose challenges on image reconstruction that limit resolution, nonetheless, the jumbophage density maps were sufficiently detailed to allow the fitting of atomic models of related capsomer proteins as hexamers and pentamers. We used the gp5 HK97 structure in the expanded capsid (PDB ID 2FT1) to fit the density of each of the maps. The resulting fits are shown as insets in Fig. 2. We used these models to partition the density maps into regions corresponding to the major capsid proteins, with remainders that we inferred to be either part of the major capsid protein (Fig. 3) or an external decoration protein (shown in yellow, orange, or red in the figure) according to the known size of the major capsid protein in the virion (Table 1; see also Fig. S1 in the supplemental material). All jumbophages under consideration here, except Bellamy, revealed strong and sizable densities inside the capsid under the five fold vertices (Fig. 3, visible [shown in yellow] in the insets). The major capsid proteins are insufficient in size to account for these density regions, and so we inferred that they arise from internal decoration. We note that the previously characterized jumbophages ΦKZ and ΦRSL1 also appear to have comparable densities under their vertices (3, 4).

10.1128/mBio.01579-17.1 FIG S1  Virion protein analysis. An SDS-polyacrylamide gel was used to measure capsid protein sizes (arrows) in mature phage particles, and these were compared to measurements from mass spectrometry (MS). Download FIG S1, PDF file, 0.1 MB. Copyright © 2017 Hua et al. 2017 Hua et al. This content is distributed under the terms of the Creative Commons Attribution 4.0 International license.

**TABLE 1:** Phages and capsid proteins^a^

Phage	Host	Major capsid protein				
		Family	Homolog(s)	Mass (kDa)	Length (no. of residues)	Locus tag
N3	*Sinorhyzobium meliloti*	T4 gp23	P-SSM2, PSSM4, Syn9	46.5, 39**	434	AVT40_gp064
PAU	*Sphingomonas paucimobilis*	T4 gp23	ZZ1, CP8, CP30A	63.4, 56.2*	585, 520*	F405_gp148
PBS1	*Bacillus subtilis*		ΦR1-37	58.9, 50**	531	PBI_PBS1_3
121Q	*Escherichia coli*	T4 gp23	PBECO 4, vB_KleM-Rak2^#^	42.2, 37**	391	PBI_121Q_603
G	*Bacillus megaterium*	HK97 gp5	Bxz1, Myrna, E3	31.2, 28.2*	282, 255*	CL58_gp027
Bellamy	*Synechococcus* sp. WH8109	T4 gp23	P-SSM2, Syn9, S-PM2	51, 45*	475	PBI_BELLAMY_133

a Phages are listed along with their hosts, major capsid protein families (inferred from sequence homologies uncovered by BLAST analysis), and the names of phages with the closest major capsid protein similarities. Jumbophage homologs are marked. The molecular masses and residue lengths of the major capsid proteins were calculated from gene sequences, and the mature, proteolyzed masses and lengths are indicated by asterisks where N-terminal sequencing data were available. Double asterisks indicate that the proteolyzed size was estimated by SDS-PAGE. Locus tag identifiers for each protein are listed.

The capsid of phage N3 is only the second example reported with a T=19 icosahedral geometry, the first being ΦM12 (24). The N3 capsid surface is relatively simple and very similar to that of ΦM12, exhibiting extra density at the vertices that may be due to an additional protein (Fig. 3A).

Phage PAU is the first example of a T=25 geometry that is different from the pseudo-T=25 geometry of adenovirus and phage PRD1 capsids, which both have trimeric capsomers in hexavalent positions and a separately encoded protein for the pentameric vertex capsomers. The capsomer bumps of PAU are more prominent than those of N3 and appear to be parts of the major capsid protein rather than a separate decoration protein. An intriguing arch of density is observed on the hexamers adjacent to each vertex, touching down at the center of each hexamer and extending to one edge in a manner that is directional around the adjacent vertex (Fig. 3B). The clear visualization of this arch after we imposed icosahedral symmetry indicated that it is organized uniformly in all PAU capsids. However, the arch does not appear to be a typical stabilizing protein, since it is not detected elsewhere on the capsid, although no other function has been attributed to it yet. Furthermore, since the PAU penton does not have an extra protein on its top, this arch could play a similar function in PAU as do the penton decoration proteins of N3, PBS1, 121Q, and Bellamy.

Phage PBS1 has the same icosahedral T=27 geometry as phages ΦKZ (3) and ΦRSL1 (4), and it also has surface features similar to ΦKZ. Interestingly, raised ridges that likely belong to the major capsid protein sit on sites of a local two-fold symmetry between capsomers, as noted for ΦKZ (Fig. 3C). The PBS1 pentamer also has extra density that might arise from an unidentified decoration protein.

Phage 121Q, with a T=28 capsid, is somewhat similar to PBS1 in that individual ridges from adjacent capsomers appear to merge across the local two-fold axes but are staggered slightly to give a zig-zag appearance, possibly due to the presence of an independent decoration protein (Fig. 3D). Furthermore, at least one other decoration protein is present at the middle of each hexon. This arrangement is unique and quite different from the two pseudo-T= 28 geometry capsids that have been reported, archaeal virus SH1 (25) and bacteriophage P23-77 (26), but which appear to belong to the internal membrane-containing family of phage PRD1 that shares architectural features with adenoviruses (27).

The largest jumbophage, phage G, ranges in diameter from 1,500 Å across the two-fold axis to 1,800 Å from vertex to vertex. Phage G has a capsid geometry of T=52 and appears to have one or possibly two decoration proteins. The major one is found at sites of local three-fold symmetry (Fig. 3E, orange density), in the manner of the gpD decoration protein trimer of phage λ (28, 29). The phage G decoration protein complex appears larger than the gpD trimer but may function similarly in stabilizing the capsid (30). We note that herpes simplex virus (HSV) and related herpesviruses have “triplex” molecules bound at similar sites of local three-fold symmetry on their nucleocapsids (31). However, even though many other aspects of HSV nucleocapsid assembly are similar to bacteriophage capsid assembly, these triplex proteins are incorporated at the earliest steps of HSV assembly (32) and remain in the mature virion, unlike the late-acting decoration proteins of phages, which only bind when the capsid is assembled and mature. The phage G pentamers include an additional density on their tops that appears to be a second separate decoration molecule (Fig. 3E, yellow portion on rendered surface map).

The prolate phage Bellamy resembles phage T4 and its relatives in having T=13 end caps and a density bound to the exterior surface in the center of each hexamer located similarly to the T4 decoration molecule, Hoc (Fig. 3F). However, the barrel of the capsid is more elongated with an additional layer of hexamers, resulting in a Q number of 24, compared to the Q=20 value for T4 (16). Bellamy also appears to have an additional decoration protein on the top of the penton (Fig. 3F), as well as a tail fiber-like cluster attached to the vertex opposite the tail (Fig. 1F). The Bellamy sample included a small number of isometric heads that package a portion of the genome and of which we have preliminary reconstructions (data not shown) that produce the same T=13 geometry as the end caps of the prolate reconstruction, much like the isometric capsids observed for T4 (33, 34).

We also assayed for the presence of an “inner body” protein structure, as seen in phage ΦKZ (7), by using bubblegrams, as it is unknown whether this is a common or even required feature of jumbo-sized capsids. In addition, an inner body would exclude some internal volume from occupation by DNA and thus drive up the local DNA density. Of the phages studied here, only 121Q demonstrated the linear bubbling pattern from electron beam damage that is consistent with a ΦKZ-like inner body (Fig. S2), indicating that such a structure is not required in large phage capsids.

10.1128/mBio.01579-17.2 FIG S2  Bubblegrams reveal inner body protein structures. Bubblegrams of the jumbophage particles, for which multiple exposures were taken by cryo-EM under low-dose conditions until radiation damage from the cumulative dose resulted in a distinctive bubble-like appearance (70). Protein in the capsid bubbles before the DNA. Only phage 121Q (d) revealed the rod-like distribution of bubbles (white arrow) that is consistent with an inner body protein structure, as seen in phage ΦKZ (7). Bubble distributions in the other phages appeared to be random. Download FIG S2, PDF file, 0.3 MB. Copyright © 2017 Hua et al. 2017 Hua et al. This content is distributed under the terms of the Creative Commons Attribution 4.0 International license.

### Jumbophage chromosomes and DNA packing densities.

**FIG 4:**
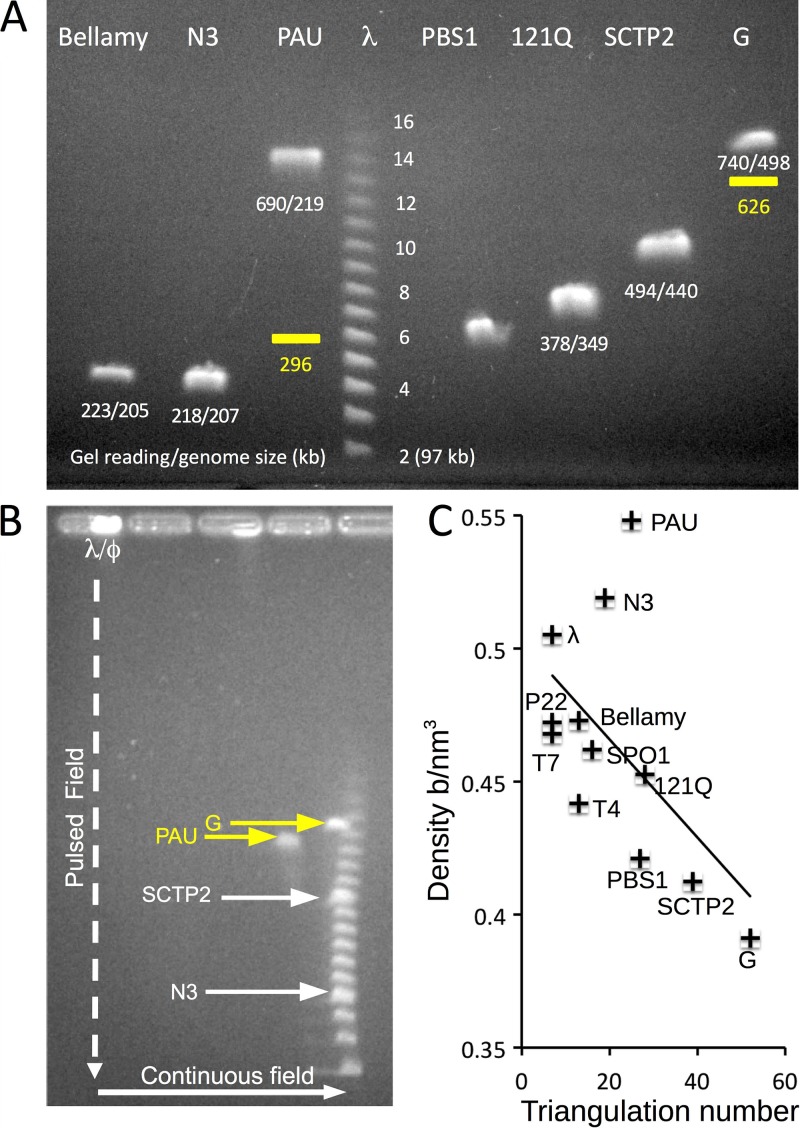
Measurement of jumbophage chromosome sizes. (A) Pulsed-field gel analysis of jumbophage DNA. Packaged DNA size was estimated by PFGE using a phage λ chromosome ladder standard; the standard shows multiples of *n* = 48.5 kbp (i.e., the *n* values [*n* × 48.5 kbp] for the ladder standard are indicated beside the ladder bands). The numbers below the jumbophage DNA bands are the apparent sizes (in kilobase pairs) of chromosomes relative to the λ ladder, followed by the sequenced genome size. All jumbophages package more than a full genome of DNA, but PAU and G appear to package considerably more. The two yellow bars indicate estimates of the true migration positions for PAU and G DNAs after applying corrections derived from 2D PFGE (see panel B) that compensated for DNA modification. (B) Two-dimensional gel of G, PAU, SCTP2, and N3 DNA. DNA from the jumbophages was loaded together with the phage λ DNA ladder in the left-most well. Pulsed-field electrophoresis was performed in the vertical direction to separate the DNA molecules based on size and mass/charge ratio. Nonpulsed electrophoresis was carried out horizontally to separate the DNA based on mass/charge ratio only. The PAU DNA migrated 26% slower than the standard, and the G DNA migrated 7% slower than the standard, indicating that these DNA molecules are modified, compared to phage N3 DNA, which migrated with the λ ladder. The magnitude of the offset allows the true DNA molecule size to be estimated, as indicated in panel A. (C) DNA packing density versus T number in jumbophages and other model phages (see Table 2). The prolate phages T4 and Bellamy were considered T*=*13.

We used pulsed-field gel electrophoresis (PFGE) (35) to measure the sizes of packaged jumbophage DNAs (chromosomes), because the mobility of such large DNA molecules is independent of size in electrophoresis with a constant field (36). The apparent chromosome sizes of Bellamy, N3, PBS1, and 121Q measured via PFGE were only slightly greater than their genome sequence lengths, as has been documented for several well-studied smaller phages (Fig. 4A; Table S2). However, the lengths of PAU and G DNA molecules appeared very much greater than their genome lengths, as measured by PFGE (Fig. 4A). We suspected that PAU and G DNA might be modified in a manner that alters their mass/charge ratio. Taking advantage of the fact that size has no effect on the mobility of very large DNA in nonpulsed electrophoresis, we conducted a 2-dimensional (2D) electrophoresis experiment in which we used PFGE for the first dimension but the second electrophoresis (at a right angle to the first) was done without pulsing. The results showed that PAU and G DNA migrated 26% and 7% slower, respectively, under electrophoresis with a constant field applied than the unmodified DNA of phage λ, presumably due to altered mass/charge ratios, indicating that these DNA molecules are indeed modified (Fig. 4B). In the same experiment, the DNA of two additional jumbophages, N3 and SCTP2, migrated with phage λ DNA, showing that these DNAs are not modified. (Phage SCTP2 was included in this experiment but it was not within the focus of this work; our findings for SCTP2 will be reported in detail elsewhere.) These values were used to calculate the “corrected” migration positions to which PAU and G DNA would migrate in PFGE in the absence of modifications (Fig. 4A, yellow bars). Using these corrected data, we calculated that the packaged DNA lengths were 296 kb for PAU and 626 kb for G; these lengths imply that the terminal redundancies for the PAU and G chromosomes are 35% and 25%, respectively (see below). We also confirmed by digestion and chromatography coupled to spectroscopy (37) that PAU has a partially modified cytosine, but the nature of the modification has not been determined (Fig. S3).

10.1128/mBio.01579-17.3 FIG S3  Separation of the deoxyribonucleosides present in bacteriophage PAU DNA. Purified plasmid (control) DNA and PAU DNA were each enzymatically digested, and the resulting deoxynucleosides were separated by reversed-phase chromatography as described in Materials and Methods. Deoxynucleosides were detected by monitoring the UV absorbance from 220 to 320 nm using a diode array detector. Absorbance at 265 nm was plotted versus time of elution, with the integrated UV spectra of each of the major peaks shown above. (A) Plasmid DNA yielded four peaks which were identified as the four normal DNA nucleosides: deoxycytidine (dC), deoxyguanosine (dG), deoxythymidine (dT), and deoxyadenosine (dA), based on their order of elution and their UV spectra (indicated in the figure). (B) PAU DNA yielded one extra late-eluting peak in addition to the four expected unmodified deoxynucleosides. The dC peak was much weaker than the dG peak, instead of the amount of dC being about equal to dG, as would be expected based on G-C base pairing in DNA; this suggested that the extra peak replaces much of the expected dC, and it is therefore labeled dC*. (C) Comparison of the UV spectra of the five deoxynucleosides found in PAU DNA. The panel emphasizes the imbalance between the dC and dG peaks and the similarities of the spectra of dC and the extra deoxynucleoside, dC*. Note that both dC and the extra nucleoside have an absorption maximum around 270 nm and a high absorption near 220 nm. These comparisons support the conclusion that the modified nucleoside in PAU is a modified form of cytosine. Download FIG S3, PDF file, 0.2 MB. Copyright © 2017 Hua et al. 2017 Hua et al. This content is distributed under the terms of the Creative Commons Attribution 4.0 International license.

We suggest that the terminal redundancy in all these jumbophages is the result of a DNA packing mechanism similar to that documented for phage T4 and others, involving “head-full” packaging into an empty capsid from a concatemer of the genome. When the head-full size, determined primarily by the volume of the capsid, is larger than the genome size, the packaged chromosome is terminally redundant. The terminal redundancy is essential for reconstituting a complete phage genome by recombination during infection.

The DNA packing density inside each of the six jumbophages, as well as inside others for which we have both the capsid structure and chromosome length, was calculated by dividing the size of the packaged DNA by the internal capsid volume obtained from the cryo-EM reconstructions (Table 2). The very largest jumbophages, such as PBS1 and G, were found to have significantly lower DNA densities than all of the others measured. This agrees with a general trend that DNA packaging density is lower for larger T number phages (Fig. 4C), although there are significant deviations from this trend. For example, the T=25 phage PAU has a much higher DNA density than the T=16 SPO1 phage and all of the T=7 phages.

**TABLE 2:** Capsids and DNA dimensions^a^

Phage	T/Q	Diam (nm)	Capsid size		Genome sequence length (kbp)	Terminal redundancy (%)	Avg density of packaged DNA (bp/nm^3^)
			Vol (×10^3^ nm^3^)	Chr DNA (kbp)			
λ	7	61	96	48.5	48.5	0	0.50
P22	7	60	90	42.5	41.7	2	0.47
T7	7	59	84	39.3	39.3	0	0.47
T4	13/20	78/108	370	175	170	3	0.47
Bellamy	13/24	78/128	464	205	190	9	0.44
SPO1	16	96	316	146	133	10	0.46
N3	19 (*l*)	107	420	218	207	5	0.52
PAU	25	112	540	296	219	35	0.55
PBS1	27	130	760	320	252	26	0.42
121Q	28 (*d*)	132	835	378	349	8	0.45
SCTP2	39	144	1,200	495	440	13	0.41
G	52 (*d*)	160	1,600	626	498	26	0.39

a The diameters were measured directly from the cryo-EM reconstructions and correspond to the inner vertex-to-vertex distances. *l* and *d* in the T/Q column indicate handedness—*laevo* and *dextro*, respectively. For the prolate phage, the first value is the inner shortest distance and the second value is the inner distance between the farthest vertices. The chromosome (Chr) DNA length of the jumbophages and P1 was determined by PFGE and 2D-PFGE. Volume is a count of internal voxels bounded by the inner surface of the capsid, determined with the icosahedron surface plugin of UCSF Chimera (62). Additional references for data on phages not detailed in this work: λ (63, 64), P22 (65), T7 (66, 67), T4 (16, 68), and SPO1 (41, 69).

## DISCUSSION

The viral capsids presented in this study represent some of the largest known, and our results complement the extensive work already done to elucidate the structures and assembly mechanisms of small- and medium-sized capsids. Larger capsids are more difficult subjects for three-dimensional structural analyses. However, with the help of direct electron camera technology, we have determined structures of these jumbophage capsids at the molecular level. For every phage (except the prolate Bellamy), we have convincingly identified the HK97 capsid protein fold and determined its organization in the capsid density maps (Fig. 2). The HK97 fold appears to be universal among dsDNA tailed phages, according to the two crystallographic structures solved to date, for HK97 (38) and T4 (39), together with a number of cryo-EM structures solved to resolutions sufficient for demonstrating consistency of capsomers (pentamers and hexamers) with the two atomic models. However, most phage capsids studied in detail to date have an icosahedral geometry of T=7, with only a few others exhibiting larger capsids, including the T=13 icosahedral phage T5 (40), T=13 prolate phage T4 (39), and several T=16 capsids, such as SPO1 (41) and Syn9 (42), which are also well-modeled as HK97 capsomers. The HK97 fold is also found in the lower domain of the herpesvirus major capsid protein (1, 43), in the major capsid proteins of archaeal virus Haloarcula sinaiiensis tailed virus 1 (44), and in the small bacterial compartments called encapsulins (45–47). Our jumbophage structures show that the HK97 fold is quite amenable to assembly into larger and more elaborate geometries. Higher-resolution structures of the jumbophage capsids and their subunits will be important for establishing the degree of similarity among capsid protein folds from large and small capsids and for revealing what may be changed to accommodate the changes in size.

In addition to the varied capsid geometries, we also observed several different arrangements of decoration proteins. Phages N3, PBS1, G, and Bellamy present extra structures on their penton vertices that possibly stabilize the pentamers, as the capsid shell around vertices is more bent than the rest of the capsid. The extra density on PAU is most intriguing, as it occurs only on peri-pentonal hexamers, where it arches from the capsomer center to one edge, obeying icosahedral symmetry but adhering poorly to quasi-equivalence. Several phages have extra density at the local three-fold axes, such as the extensive “mushrooms” on the phage G surface, with a morphology that resembles the trimer of protein gpD, which stabilizes the capsid of phage λ. It is not yet known whether the jumbophage decoration proteins are used to stabilize these large capsids, and so experiments, such as the deletion of decoration protein genes, will be needed to understand the roles of these proteins in jumbophage assembly. (It would then be useful to establish a molecular genetic analysis system for at least one jumbophage that would allow experiments like this to be done.) Every icosahedral jumbophage examined here has additional density on the interior side of the pentameric vertices. The function of these structures is unknown. One possibility is that they reinforce the pentons, which may be a constitutively weak part of the capsid (48). These subpenton densities could also play a role in directing the correct assembly of the capsid.

The jumbophage capsids are unlike those of other large capsids found to date. Members of the adenovirus fold family have pseudo-T numbers ranging from 25 to 219, but hexavalent sites are occupied by a trimer of the “hexon” protein while the pentavalent positions hold a pentamer of a different protein that, in some cases, binds a trimeric fiber molecule (49). The intercapsomer spacing for capsids with the trimeric adenovirus architecture is smaller than for capsids constructed with hexamers, and consequently the capsid sizes are not as large as for jumbophages of equivalent T numbers. For example, the pseudo-T=25 adenovirus capsid diameter (excluding the penton base fibers) is 900 Å, whereas the diameter of PAU (T=25) is 1,160 to 1,280 Å (across the two-fold and five-fold axes, respectively). In addition, the large algal virus capsids of Chilo iridescent virus (T=147), Paramecium bursaria chlorella virus type 1 (T=169) (18), and Phaeocystis pouchetii virus PpV01 (T=219) (20), which all share the adenovirus capsid fold, are only slightly larger, with 1,900- to 2,200-Å diameters across the five-fold axis, than phage G at 1,800 Å, despite its lesser triangulation number (T=52). However, the *Mimivirus* capsid is much larger at ~5,000-Å diameter, and the resolutions achieved with cryo-EM-based image reconstructions only provided rough estimates of the triangulation number (T=972 to 1,200). Indications are that the subunit fold also belongs to the adenovirus/PRD1 family (22). Whether HK97-like capsids extend beyond the dimensions of phage G remains to be seen, but as with the large algal viruses and the *Mimiviridae* family, any such viruses may simply have been overlooked.

A question that arises from observing these new HK97-like jumbophages is how assembly of a core structural motif can be guided into many geometries that are highly specific for each virus. In the case of smaller viruses, including HK97 itself, the capsid protein contains all the information for self-assembly into a specific icosahedral geometry (50, 51). Larger capsids tend to utilize one or more scaffolding proteins that coassemble with the major capsid protein or preassemble into a core on which the capsid protein assembles. Although the phage data presented here do not explain how this process works in each case, we may reasonably expect that the HK97-like fold has sufficient flexibility to adjust to many subtle geometric variations, possibly through the addition of extra domains or the alteration of capsomer interfaces.

Perhaps a more compelling question is why viruses of a common capsid protein fold developed into related structures with such a wide variation in dimensions. In other words, if the progenitor was a simple T=1 (or T=small) icosahedron, what provoked expansion into the T=52 behemoth that is phage G, with its correspondingly huge genome? We posit here a genetic ratchet mechanism as a model for capsid size expansion that combines observations on capsid size as affected by mutations in the major capsid or scaffold proteins together with the head-full DNA packaging process and recombination events documented in many viral genomes.

The size of the genome and the size of the capsid are coupled in the sense that a larger genome requires a larger capsid to accommodate it, and a larger capsid is able to accommodate a larger genome. If a genome were to become significantly larger, for example, by insertion of a novel DNA sequence into the genome through horizontal gene transfer, the resulting virus would not be viable because the head-full packaging mechanism of the unaltered capsid could not accommodate the newly expanded genome. On the other hand, if the gene of the major capsid protein acquired a mutation that resulted in assembly of a larger capsid, the resulting virus would still be viable, differing only in having a larger terminal redundancy. Such a virus could revert by back mutation to the original capsid size, since that capsid size would still accommodate the unaltered genome. If instead the genome acquired a substantial amount of new DNA by horizontal gene transfer, or perhaps by tandem duplication of genes already present, the genome could still be successfully packaged into the larger capsid by the head-full packaging mechanism. However, reversion to the original smaller capsid size after new DNA has been introduced into the genome would be unlikely, since it would require deletion of the newly acquired segment(s) of DNA before the capsid gene mutation causing the larger capsid size could revert. Such a reversion to a smaller capsid size would be even less likely if the inserted DNA had acquired, by mutation or by ancestry, a function that provided a selective benefit to the phage. By the mechanisms outlined, reversion to a smaller capsid size would be strongly disfavored and the new larger capsid architecture would thus become locked in as by a ratchet. In this model, capsids would tend to increase in size, although presumably at some metabolic cost (more capsid protein to make and more DNA to package per capsid) that might be avoided by capsids that refrain from undergoing ratcheting.

Mutations affecting capsid size are well-known and include T4 hexamer protein gene mutants that assemble isometric T=13 particles (33, 34) in addition to prolate T=13/Q=20 particles (16). It may be that the same point mutation in reverse would be equally likely and would reshape a T=13 icosahedral capsid into a much larger prolate capsid, and indeed, other T4 mutations produce wildly elongated T4 heads or even polyheads that are effectively endless tubes (52). In addition to the size variant mutations, a few phages, such as P1, naturally produce icosahedral capsids of several sizes that all package DNA and bind tails (53). Thus, modifications of capsid size appear far from uncommon and may well serve in our genetic ratchet mechanism. However, while phages T4 and P1 demonstrate that alternative capsid sizes are accessible, productive ratcheting of the capsid size probably also requires good control and fidelity for the new structure.

If the jumbophages have in fact acquired their large capsid and genome sizes by the ratcheting mechanism we propose here, then some fraction of their genomes should have been acquired since the capsid proteins learned how to make a larger capsid. At this point, we cannot identify which genes might fall into this category, since we do not know the genome content of any of the putative precursor phages from which the jumbophages were derived. However, we note that the majority of putative proteins encoded in a typical jumbophage genome make no believable BLAST hits with anything currently in the sequence databases. These are certainly candidates for genes recently acquired through horizontal transfer.

A possibly more compelling case can be made that one way the jumbophage genomes have expanded is through tandem duplication of preexisting genes. In both phage G and phage 121Q, we found a tandem array of more than 30 very similar genes that we interpreted to be the result of tandem duplication.

The bacteriophage packs its DNA to an extremely high density that is only observed in crystalline DNA (54). Viral DNA packaging in jumbophages is likely to be similar to the head-full mode in phages like T4. Head-full-sized chromosomes are sequentially packaged from the concatameric DNA without sequence specificity at the two ends. Terminal redundancy in the chromosomal DNA ensures that individual virions possess all the genes essential for viability. Since the capsid structures, chromosome DNA lengths, and genome sequences of jumbophages are available, we naturally ask the question if the terminal redundancy and DNA packing density is related to the capsid size. The terminal redundancy of well-known smaller bacteriophages, like T4, λ, P22, and SPP1, is mostly under 4%. The terminal redundancy of the jumbophages is mostly higher, ranging from 5% in N3 to 35% in PAU. It is conceivable that smaller phages, especially the numerous T=7 phages, cannot afford a large piece of redundant DNA sequence because the limited capsid space must be reserved for the complete set of genes required for viability. However, all six jumbophages adapt to large sequence redundancy, suggesting that the large terminal redundancy may provide some common advantages. Alternatively, a relatively large terminal redundancy may simply be a result of a relatively recent increase in capsid size.

The DNA of PAU and G exhibited slower migration in electrophoresis than regular DNA (much slower in the case of PAU). We showed, using a 2D gel approach that combined both pulsed-field and constant-field electrophoresis, that at least one of the nucleotides of both PAU and G DNA is modified and has either lost a negative charge or acquired a positive one. Such DNA modifications have occurred in many bacteriophages, and while some of these have been catalogued previously (55), their potential functions and evolutionary advantages remain open and exciting questions.

Additional jumbophage capsid structures will form the basis for understanding the mechanisms and component proteins of virion assembly and size determination. In combination, analysis of genome sizes and comparison with the quantity of DNA packaged will offer insights into how capsids become locked into larger sizes once mutations render them capable of expansion. We expect this investigation of capsid and genome coevolution to reveal how viruses acquire novel capabilities and what adaptations are made to stabilize very large capsids.

## MATERIALS AND METHODS

### Phage preparation.

Phages PAU, PBS1, and 121Q and their hosts were obtained from the Felix d’Hérelle Phage Culture Collection (Université Laval, Québec, Canada) (http://www.phage.ulaval.ca); phage G was obtained from the American Type Culture Collection; N3 and its host were from the collection of Sharon Long and were supplied to us by Valerie Oke; Bellamy was isolated by enrichment of water from the harbor of Jamestown, RI.

High-titer phage stocks of N3, PAU, PBS1, and 121Q were produced by mixing approximately 10,000 PFU of the phage, 200 µl of overnight host cells (Table 1) growing in LB, and 10 ml warm LB containing 0.4% agar, 1 mM CaCl_2_, and 5 mM MgSO_4_. The mixture was poured onto a 150-mm by 15-mm fresh LB agar plate and incubated overnight at 30°C for N3 and PAU or at 37°C for PBS1 and 121Q. Phages were extracted from the top agar by mixing the agar with an equal volume of 10 mM MgSO_4_, 10 mM Tris (pH 7.9) buffer (TM buffer), followed by vigorous vortexing. The insoluble materials were removed by centrifugation in a Beckman JA-50.250 rotor at 8,000 rpm for 10 min. Phages in the supernatant were pelleted in a Beckman type 45Ti rotor at 4°C, 35,000 rpm for 1 h and then suspended in buffer. Phages PAU and PBS1 were further purified using CsCl step gradients (5 ml of sample layered on 3 ml 30% glycerol, 2 ml 1.4-g/ml CsCl, and 2 ml 1.6-g/ml CsCl, with all layers in TM buffer) run in a Beckman SW41 rotor at 30,000 rpm, 18°C, for 90 min. Phages N3 and 121Q are sensitive to concentrated CsCl solution, so they were purified by velocity sedimentation in 10% to 45% sucrose gradients in TM buffer in a Beckman SW41 rotor at 30,000 rpm, 18°C, for 60 min. The visible phage bands in both gradient types were collected and dialyzed against TM buffer overnight, before being examined by cryo-EM.

A high-titer sample of Bellamy suitable for cryo-EM was obtained by growing a 9-liter culture of *Synechococcus* sp. WH8109 in artificial seawater medium to mid-log phase and adding virus particles at a multiplicity of infection of 0.1. Infection and lysis occurred over several days, during which the cultures were maintained at room temperature and constant low-level illumination at ~6 to 8,000 lx/m^2^/s provided by cool white fluorescent bulbs. Lysis was judged to be complete when the cultures became clear and bright green. Cell debris was removed by centrifugation at 8,000 rpm for 10 min in a Beckman JA-10 rotor and further clarified by sequential filtration through filter paper and a 0.22-µm filter. NaCl was added to 0.5 M, followed by 15% (wt/vol) polyethylene glycol 8000. The phage precipitate was collected by centrifugation at 9,000 rpm for 15 min in a Beckman JA-10 rotor. The precipitate was resuspended in approximately 40 ml of 0.5 M NaCl, 50 mM MgSO_4_, and 10 mM Tris (pH 8), and approximately 8 g of CsCl was added to every 10 ml of solution. The CsCl-phage solution was then loaded into 13.9-ml heat-sealed tubes and centrifuged in a Beckman type 70Ti rotor for 16 h, 18°C, at 28,000 rpm. Visible phage bands were removed using a needle and dialyzed against 100 mM NaCl, 50 mM MgSO_4_, 10 mM Tris-HCl (pH 8) buffer for cryo-EM.

Phage G was propagated in *B. megaterium* grown in 2.8-liter Fernbach flasks at 37°C with vigorous aeration. One liter of LB medium was inoculated with 10 ml of a saturated *B. megaterium* culture and grown for 2 h, at which time the temperature was lowered to 30°C and 10^9^ PFU of phage G was added. The culture was incubated for a further ~5 to 6 h until lysis was observed. Cell debris and unlysed cells were removed by two 30-min centrifugations at 680 × *g* (2,000 rpm; Sorval GS3 rotor). The clear supernatant was centrifuged for 2 h at 9,500 × *g* (7,500 rpm; Sorval GS3 rotor) to collect the phage. Pellets containing phage were covered with G phage buffer (10 mM MgSO_4_, 10 mM Tris-HCl [pH 7.5]), suspended by gentle rocking overnight at 4°C, and further purified by velocity sedimentation in 10 to 35% (wt/vol) sucrose gradients at 4°C in the same buffer, using a Beckman SW28 rotor run for 40 min at 14,300 rpm. The phage band was extracted and dialyzed against G phage buffer to remove sucrose. Lysate titers were determined on LB plates by using a soft agar overlay containing 40 μl of plating bacteria, 100 μl of diluted phage, and 3.5 ml of top agarose (LB plus 1 mM CaCl_2_ plus 0.15% agarose). Plates were incubated at 30°C overnight and for 1 day at room temperature. Plating bacteria were prepared by growing *B. megaterium* to mid-log phase in LB at 37°C with vigorous aeration, followed by further incubation without aeration overnight at room temperature.

### Cryo-electron microscopy and image reconstruction.

Purified phage samples were prepared for cryo-EM and imaged according to standard methods (56). Briefly, 3.5 μl of sample was pipetted onto freshly glow-discharged R2/1 Quantifoil grids (Quantifoil Micro Tools GmbH, Jena, Germany) and plunge-frozen into liquid ethane-propane (50:50 mix) cooled in a liquid nitrogen bath (57) by using an Vitrobot Mark III apparatus (FEI, Hillsboro, OR). The grids were transferred to either an FEI Tecnai T20 FEG microscope operating at 200 kV (for Bellamy), an FEI Titan Krios microscope operating at 300 kV (for 121Q), or an FEI Polara microscope operating at 300 kV (all other phages). For Bellamy, images were collected at 29,000× magnification on SO-163 film (Kodak, Rochester, NY) and digitized on a Super CoolScan 9000 scanner (Nikon, Tokyo) at a sampling rate of 6.35 μm/pixel, corresponding to 2.17 Å/pixel at the sample. For PAU, images were collected at ×38,000 magnification on SO-163 film (Kodak, Rochester, NY) and digitized as described above, corresponding to 1.63 Å/pixels at the sample. For all other phages, we collected images on an FEI Falcon 2 direct electron detector under automated control of the FEI EPU software. For the Krios images, the nominal magnification of ×75,000 and postcolumn magnification of ×1.6 yielded calibrated pixels of 1.08 Å at the sample. For the Polara, the nominal magnification of ×78,000 and postcolumn magnification of ×1.4 yielded calibrated pixels of 1.37 Å at the sample. The total dose per exposure was estimated to be ~30 e^−^/Å^2^. Phage particles were selected using the x3dpreprocess software (58), and defocus/astigmatism values were estimated using ctffind3 (Mindell, Grigorieff 2003). An icosahedrally averaged map of each sample was calculated using AUTO3DEM (59) by refining an initial model generated from the images using the random model procedure (60). For the prolate phage Bellamy, particle selection in x3dpreprocess was modified to allow an extended circle mask (i.e., two circles connected by tangents), and each particle was rotated to bring the tail ends in approximate mutual alignment. The reconstruction was done using EMAN (61) with the imposition of five-fold symmetry about the tail axis. UCSF Chimera (62) was used to visualize and analyze the density maps. Bubblegrams (7) were recorded with consecutive 1-s exposures onto a Gatan Ultrascan 4000 charge-coupled-device camera until the formation of bubbles was observed.

### DNA analysis.

PFGE experiments were conducted using a Bio-Rad CHEF-DR III pulsed field electrophoresis system according to the manufacturer’s instructions. Phage chromosome DNA samples were embedded in agarose by combining 1 × 10^7^ phage with warm agarose (1%) in water to a total volume of 60 µl and then transferring the mixture into plug molds to solidify. The phage-agarose plugs were soaked in 0.5 ml buffer containing 0.5 M EDTA, 0.01 M Tris (pH 9.5), 1% *N*-laurylsarcosine, and 400 µg/ml proteinase K and incubated in a 60°C water bath overnight. The phage-agarose plugs were soaked in 0.5× Tris-borate-EDTA (TBE) gel buffer for 1 h before being inserted into the wells of a 1% agarose gel. Pulsed-field electrophoresis was carried out under conditions of 0.5× TBE buffer, 14°C, 6 V/cm voltage, 50- to 90-s switch time, 22-h running time, 120° angle, and with the NEB lambda ladder marker. The pulsed-field gel measurements had relative standard deviations ranging from 0.02 to 0.05 for any individual phage.

In the 2D gel electrophoresis experiment, the DNA of G, PAU, N3, SCTP2, and the lambda marker were mixed in a single gel plug. (SCTP2 DNA was present in this experiment but was not within the focus of this work; the results of that experiment will be reported elsewhere.) The gel plug was loaded in the well at the upper left, and the DNA mixture was electrophoresed in a pulsed field using the previous PFGE settings. A second electrophoresis without pulsing was then applied perpendicular to the initial PFGE in a Hoefer HE99X large agarose gel box in 0.5× TBE at 110 V for 8 h. The second-dimension migration distances were reproducible to better than ±5%.

### DNA composition analysis.

Analysis of phage DNA base composition followed an established method (37). Samples of purified DNA were digested sequentially with DNase I, nuclease P1, and bacterial alkaline phosphatase, and the resulting deoxynucleosides were separated by chromatography on a 3.9- by 300-mm µBondapak C_18_ column (Waters, Milford, MA). Chromatography was run at 1 ml/min and 45°C using 2.5% (vol/vol) methanol in 0.01 M ammonium phosphate (pH 5.3) for 30 min followed by 8% (vol/vol) methanol in 0.01 M ammonium phosphate (pH 5.1) for 40 min (37). We enhanced the earlier method by continuously monitoring the UV absorbance spectra from 220 to 320 nm at 5-s intervals during elution, using a Waters 990 photodiode array detector (Waters, Milford, MA). The UV spectra were used to help classify the nucleoside peaks.

### Data availability.

Details of the phage image reconstructions are provided in Table S1; the table also includes the identification numbers for density maps deposited with the EM DataBank (http://emdatabank.org).

10.1128/mBio.01579-17.4 TABLE S1  Details of reconstructions. Data sets of images were collected on an FEI cryo-electron microscope equipped with film or a Falcon 2 direct electron-detecting camera. Resolution was assessed at the spatial frequency where the Fourier shell correlation (FSC) calculated between independent half-data set maps reached 0.5 (71). Icosahedral symmetry was imposed on the first 5 data sets, but for phage Bellamy only five-fold symmetry about the longitudinal axis was imposed. Density maps were deposited at the EM DataBank [http://emdatabank.org] with the IDs listed. *, since the resolution was assessed with FSC of 0.5 and only 344 particles were used for the final reconstruction, the value obtained is not representative of the map quality. Download TABLE S1, PDF file, 0.1 MB. Copyright © 2017 Hua et al. 2017 Hua et al. This content is distributed under the terms of the Creative Commons Attribution 4.0 International license.

10.1128/mBio.01579-17.5 TABLE S2  Accession numbers for jumbophage genomes. Download TABLE S2, PDF file, 0.1 MB. Copyright © 2017 Hua et al. 2017 Hua et al. This content is distributed under the terms of the Creative Commons Attribution 4.0 International license.
