# Supplementary material for: Capsids and Genomes of Jumbo-Sized Bacteriophages Reveal the Evolutionary Reach of the HK97 Fold
Source: mBio. 2017 Oct 17;8(5):e01579-17. doi: 10.1128/mBio.01579-17 (PMC5646251; doi:10.1128/mBio.01579-17)
Supplement: FIG S1 [file mbo005173536sf1.pdf]

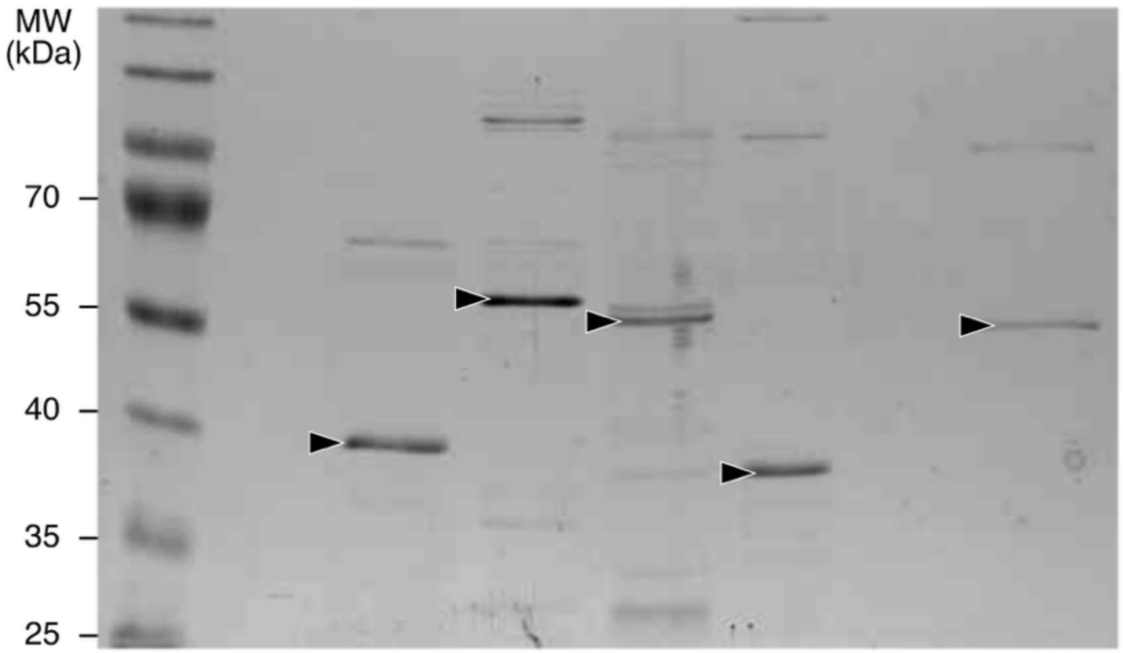

	N3	PAU	PBS1	121Q	G	Bellamy
MW from gel (kDa):	39	58	55	37	–	54
MW from MS (kDa):	–	56	50	–	28	45

Supplementary Figure 1. Virion protein analysis. An SDS polyacrylamide gel was used to measure capsid protein size (arrows) in mature phage particles, and these are compared to measurements from mass spectrometry (MS).
