# Supplementary material for: Capsids and Genomes of Jumbo-Sized Bacteriophages Reveal the Evolutionary Reach of the HK97 Fold
Source: mBio. 2017 Oct 17;8(5):e01579-17. doi: 10.1128/mBio.01579-17 (PMC5646251; doi:10.1128/mBio.01579-17)
Supplement: FIG S2 [file mbo005173536sf2.pdf]

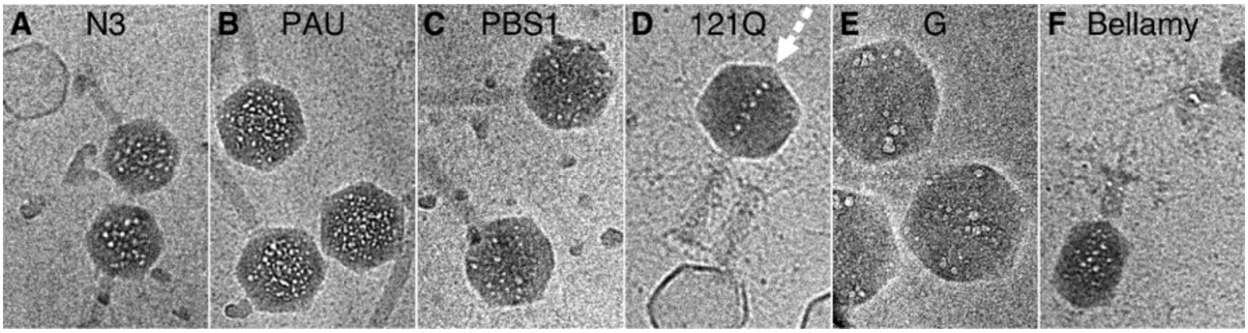

Supplementary Figure 2. “Bubblegrams” reveal inner-body protein structures. “B ubblegrams” of the jumbophage particles where multiple exposures are taken in cryo-EM low-dose conditions until radiation damage from the cumulative dose results in a distinctive bubble-like appearance (1). Protein in the capsid bubbles before the DNA. Only phage 121Q (D) reveals the rod-like distribution of bubbles (white arrow) that is consistent with an “inner body” protein structure, as seen in phage phiKZ (2). Bubble distributions in the other phages appear to be random.
