# Supplementary material for: Capsids and Genomes of Jumbo-Sized Bacteriophages Reveal the Evolutionary Reach of the HK97 Fold
Source: mBio. 2017 Oct 17;8(5):e01579-17. doi: 10.1128/mBio.01579-17 (PMC5646251; doi:10.1128/mBio.01579-17)
Supplement: FIG S3 [file mbo005173536sf3.pdf]

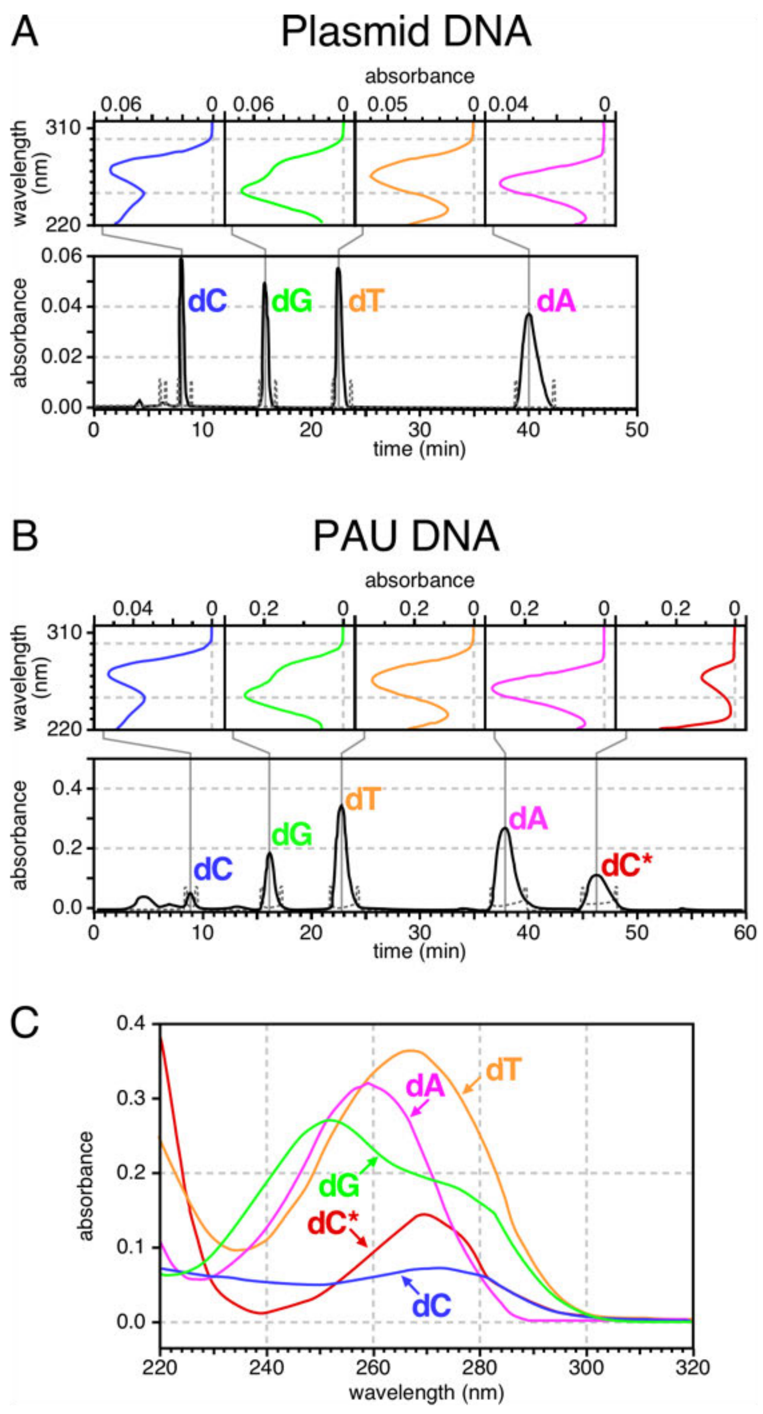

Supplementary Figure 3: Separation of the deoxyribonucleosides present in bacteriophage PAU DNA. Purified plasmid (control) DNA and PAU DNA were each enzymatically digested and the resulting deoxynucleosides were separated by reversed phase chromatography as described in Materials and Methods. Deoxynucleosides were detected by monitoring the UV absorbance from 220 to 320 nm using a diode array detector. In panels A and B, the absorbance at 265 nm is plotted versus time of elution, and the integrated UV spectra of each of the major peaks are shown above. (A**)** Plasmid DNA yielded four peaks which were identified as the four normal

DNA nucleosides: deoxycytidine (dC), deoxyguanosine (dG), deoxythymidine (dT), and deoxyadenosine (dA) based on their order of elution and their UV spectra as indicated in the figure. (B) PAU DNA yielded one extra late-eluting peak in addition to the four expected unmodified deoxynucleosides. The dC peak was much weaker than the dG peak, instead of dC being about equal in amount to dG, as would be expected due to G-C base pairing in DNA, suggesting that the extra peak replaces much of the expected dC and is therefore labeled dC*. (C) Comparison of the UV spectra of the five deoxynucleosides found in PAU DNA. The panel emphasizes the imbalance between the dC and dG peaks, and the similarity of the spectra of dC and the extra deoxynucleoside, dC*. Note that both dC and the extra nucleoside have an absorption maximum at around 270 nm and a high absorption near 220 nm. These comparisons support the conclusion that the modified nucleoside in PAU is a modified form of cytosine.
