# Supplementary material for: Capsids and Genomes of Jumbo-Sized Bacteriophages Reveal the Evolutionary Reach of the HK97 Fold
Source: mBio. 2017 Oct 17;8(5):e01579-17. doi: 10.1128/mBio.01579-17 (PMC5646251; doi:10.1128/mBio.01579-17)
Supplement: TABLE S1 [file mbo005173536st1.pdf]

Phage	Microscope & camera	Micrograph count	Particle counts		Resolution (Å)	EMD ID
			picked	final map		
N3	Polara, F2	1352	1914	1531	9	8487
PAU	Polara, film	177	10348	9260	9	8488
PBS1	Polara, F2	3000	2000	2000	10	8486
121Q	Krios, F2	3761	6242	5566	9	8485
G	Polara, F2	204	344	344	15*	8484
Bellamy	TF20, film	56	10600	10600	~30	8489

Supplementary Table 1. Details of reconstructions. Data sets of images were collected on FEI cryo-electron microscopes, as indicated, equipped with film or the Falcon 2 (F2) direct electron detecting camera. Resolutions was assessed at the spatial frequency where the Fourier Shell Correlation calculated between independent half-dataset maps reaches 0.5 (13). Icosahedral symmetry was imposed on the first 5 datasets, but for Bellamy only 5-fold symmetry about the longitudinal axis was imposed. Density maps were deposited at the EM DataBank (http://emdatabank.org) with the IDs listed. *Since the resolution has been assessed by FSC=0.5 and only 344 particles have been used for the final reconstruction, the value obtained is not representative of the map quality.
