# Supplementary material for: Capsids and Genomes of Jumbo-Sized Bacteriophages Reveal the Evolutionary Reach of the HK97 Fold
Source: mBio. 2017 Oct 17;8(5):e01579-17. doi: 10.1128/mBio.01579-17 (PMC5646251; doi:10.1128/mBio.01579-17)
Supplement: TABLE S2 [file mbo005173536st2.pdf]

Phage	Genome size	GenBank Accession number
N3	206,713	NC_028945
PAU	219,372	NC_019521
PBS1	252,179	MF360957
121Q	348,532	NC_025447
G	497,513	NC_023719
Bellamy	204,934	MF351863
SCTP2	440,001	MF360958

Supplementary Table 2. Accession numbers for jumbophage genomes.

Supplementary References

1. **Wu W, Thomas JA, Cheng N, Black LW, Steven AC.** 2012. Bubblegrams reveal the inner body of bacteriophage phiKZ. Science **335:**182.

2. **Thomas JA, Weintraub ST, Wu W, Winkler DC, Cheng N, Steven AC, Black LW.** 2012. Extensive proteolysis of head and inner body proteins by a morphogenetic protease in the giant Pseudomonas aeruginosa phage phiKZ. Molecular microbiology **84:**324-339.

3. **van Heel M.** 1987. Similarity measures between images. Ultramicroscopy **48:**95-100.
